# Penile Fracture With Urethral Bleeding: A Urological Emergency

**DOI:** 10.7759/cureus.31373

**Published:** 2022-11-11

**Authors:** Harshil Krishnani, Ashish Anjankar, Gauri Kakar, Samriddhi Sharma, Abhishek Kumar

**Affiliations:** 1 Medicine, Jawaharlal Nehru Medical College, Datta Meghe Institute of Medical Sciences, Wardha, IND; 2 Biochemistry, Jawaharlal Nehru Medical College, Datta Meghe Institute of Medical Sciences, Wardha, IND; 3 Medicine and Surgery, Jawaharlal Nehru Medical College, Datta Meghe Institute of Medical Sciences, Wardha, IND; 4 Medical Education, Jawaharlal Nehru Medical College, Datta Meghe Institute of Medical Sciences, Wardha, IND

**Keywords:** proline 2.0 suture, eggplant deformity, erection, urethral bleeding, penile fracture

## Abstract

Erection transforms the penis from a safe, flaccid organ to a susceptible one. During an erection, the thick tunica albuginea thins and becomes fracturable. Penile fracture (PF) is a very uncommon ailment produced by a blow to the erect penis. Unphysiological bending of the erect penis during sexual activity or masturbation is the most often reported mechanism of trauma. The penis is made up of three columns of erectile tissue: one ventral corpus spongiosum and two dorsolateral corpora cavernosa, each enclosed by the tunica albuginea. The tunica albuginea is a bilaminar structure composed of collagen and elastin. The outer layer of the tunica determines its strength and thickness. It varies in several locations across the shaft and is thinnest ventrally. It has high tensile strength and can sustain rupture at intra-cavernous pressures of up to 1500 mmHg. The tunica albuginea's thickness decreases from 2 mm to 0.25 mm while the penis is erect, and a trauma-induced rise in intracorporeal pressure during an erection might easily cause rupture. PF with urethral bleeding is a very rare urological emergency. One of the common causes of PF includes vigorous sexual intercourse. Symptoms include a cracking, snapping, or clicking sound followed by an instantaneous detumescence. Additionally, the penis may exhibit acute discomfort, significant ecchymosis, rapid swelling, and noticeable eggplant deformity. This is a case report of a 30-year-old male who presented with a history of penile swelling and ecchymosis during sexual intercourse. There was blood spotted at the urethral meatus. A retrograde urethrogram showed complete disruption at the proximal third of the urethra. The patient was immediately taken for surgery, and extensive exploration was done. There was a significant defect of the tunica albuginea of the corpora cavernosa on the ventral side of the penis. Along with the defect and the PF, there was a sizeable urethral injury as well. The defect of the tunica albuginea of the corpora cavernosa was repaired with a Prolene 2.0 suture (Johnson & Johnson, New Brunswick, New Jersey, United States), and urethral reconstruction was done with Vicryl 3.0 interrupted suture (Johnson & Johnson, New Brunswick, New Jersey, United States) over a 14-Foley catheter. Ultrasonography (USG) can be used to assess patients who have suffered penile injuries as well as to determine the sort of incision that is necessary, saving time by preventing needless waiting. This instance emphasises the value of early diagnosis in cases of unique presentation and early surgical treatment for improved results.

## Introduction

Penile fracture (PF), a rare urological emergency, must always be treated immediately. In 9% to 20% of cases, urethral trauma may be present. PF and urethral injury are related, particularly in a suggestive physical examination and medical history findings such as severe urine retention and urethral bleeding. Immediate primary surgery is the safest and most efficient method for treating urethral injury and PF simultaneously [1]. The most typical cause of injury to the erect penis was sexual intercourse. PF can occur when the penis slides out of the vagina during intense sexual intercourse and strikes the symphysis pubis or perineum. The most frequent clinical signs were a cracking, snapping, or clicking sound followed by an instantaneous detumescence. Additionally, the penis may exhibit acute discomfort, significant ecchymosis, rapid swelling, and noticeable deformity. Methods may include taking a history, performing a physical examination, and imaging. Early surgical diagnosis and treatment are crucial for the best results [2]. When there is a fracture of the corpus cavernosum, a hematoma forms over the injured area, which causes the penis to deviate from the injured area, giving patients the traditional "eggplant deformity" appearance [3]. Although PF is a rare urogenital injury, it is crucial to be aware of it. Most PFs result from direct damage during sexual contact. The cavernosal experiences greater pressure following direct damage to an erect penis. The tunica albuginea ruptures as a result of the increasing pressure. PF treatment should not be delayed as it may result in long-lasting sexual and anatomical problems. In urology, a PF is regarded as an emergency [4].

Surgery must be performed immediately. Typically, a catheter serves to recognize the urethral route and protect it from being injured by the dissection performed to treat the PF. In this case report, the patient had a PF and urethral bleeding, which required urgent surgical attention and a Foley catheter to stop the bleeding. A Foley catheter is a flexible tube that professional medical inserts through the urethra and into the bladder to drain urine [1,3].

## Case presentation

**Figure 1:**
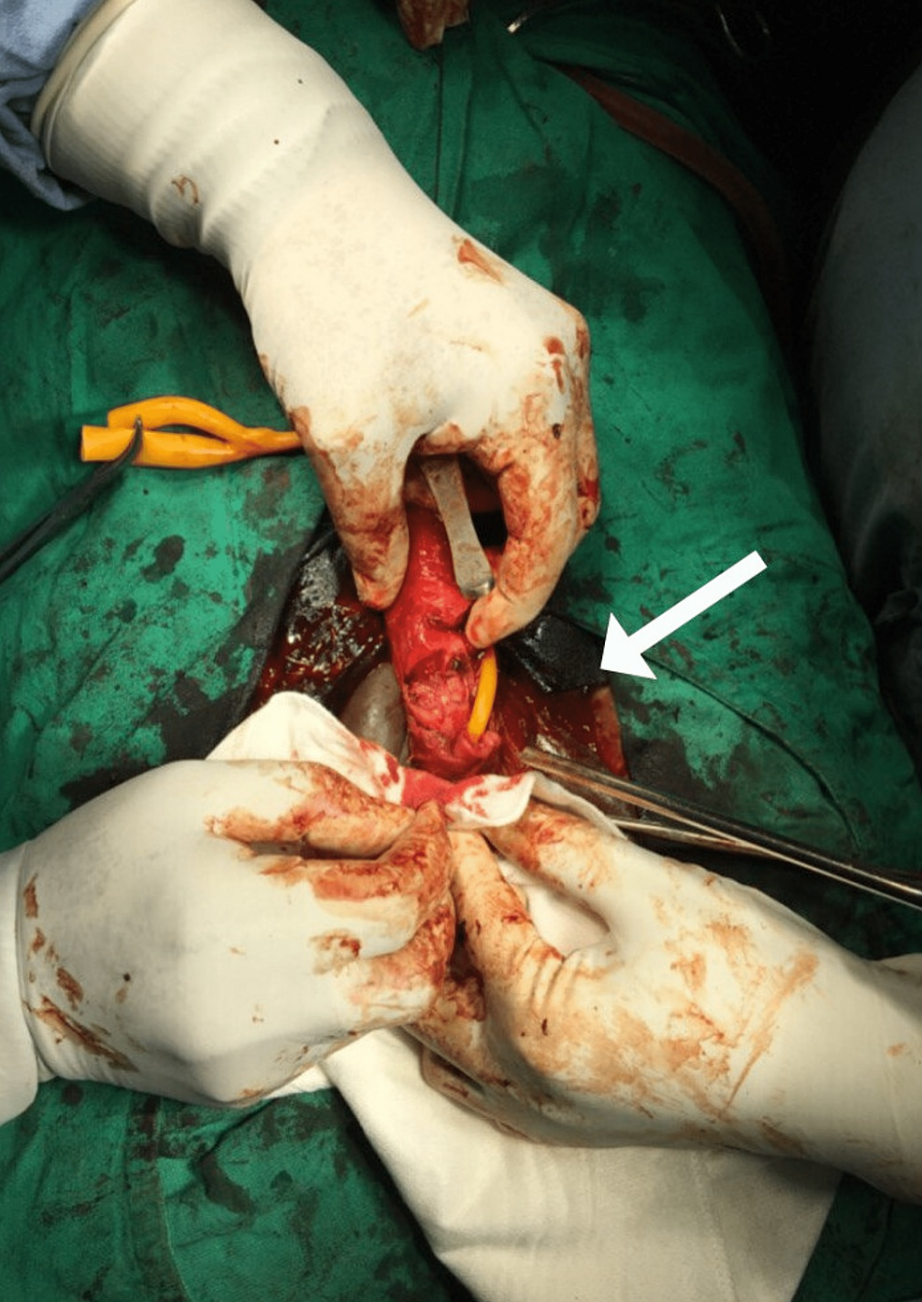
Large disruption of the corpus spongiosum

A 30-year-old male patient presented to the emergency department with a history of penile swelling and ecchymosis during sexual intercourse in a "woman-on-top" position. He gave a history of clicking/snapping sound followed by abruptly sharp pain. He immediately lost his erection after experiencing pain. He had blood coming out of the tip of his penis. Moving and urinating were some of the factors that aggravated the pain. There was no history of hypertension, diabetes mellitus, or prior surgeries. He denied having any sexual disorders or using phosphodiesterase-5 (PDE-5) inhibitors in the past. On examination, eggplant deformity of the scrotum and penis was also noted. There was blood spotted at the urethral meatus. A retrograde urethrogram showed complete disruption at the proximal third of the urethra. All this confirmed the diagnosis of PF with urethral bleeding. All other investigations such as complete blood count (CBC), kidney function test (KFT), and urine analysis were normal. Urgent surgical treatment was required. The patient was immediately taken up for surgery. Extensive exploration was done using subcoronal degloving incision. This incision was used due to its better long-term recovery and because it is better cosmetically. On exploration, there was a large defect of the tunica albuginea of the corpora cavernosa on the ventral side of the penis. Along with the defect and the PF, there was a large disruption of the corpus spongiosum as shown in Figure 1.

**Figure 2:**
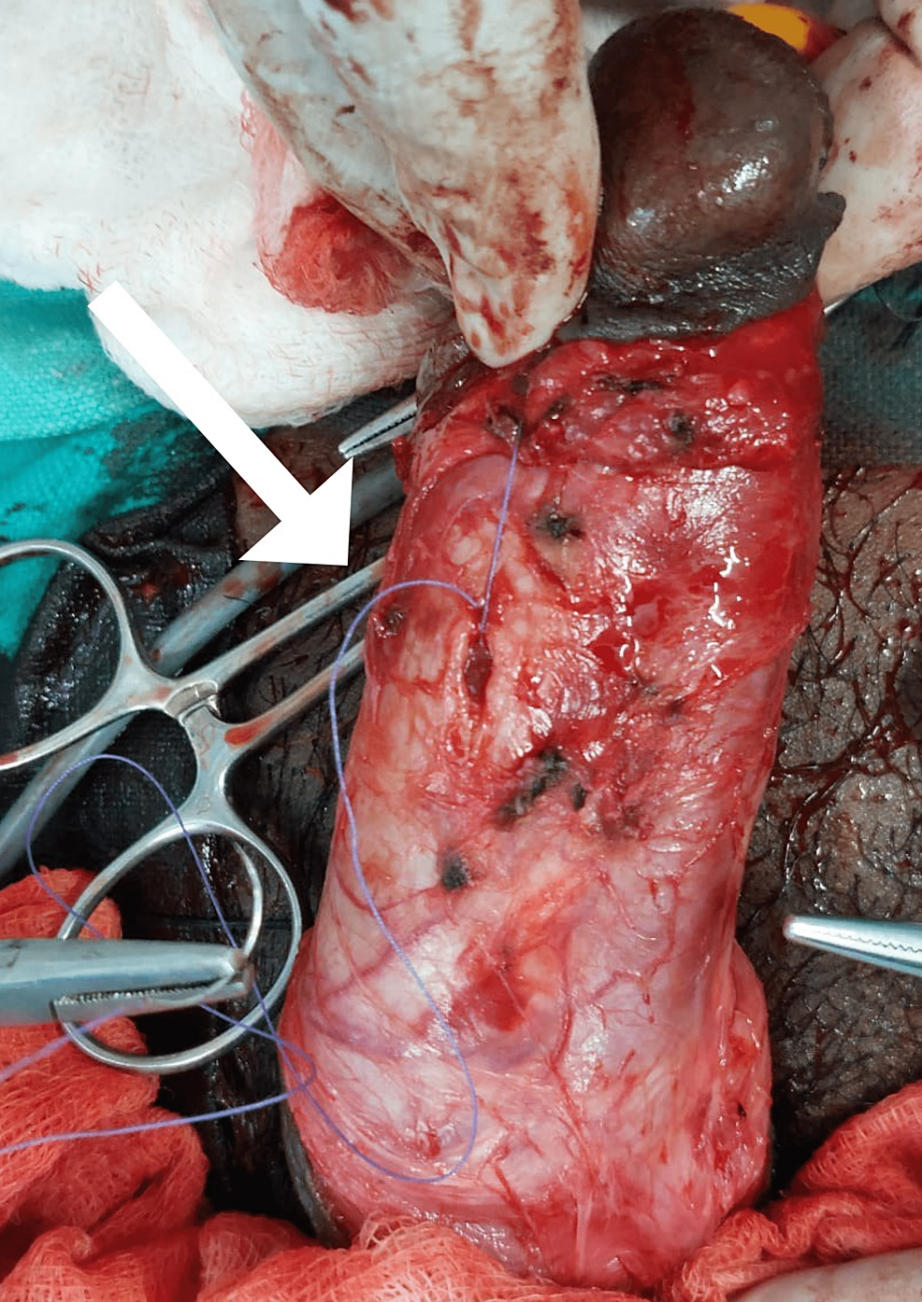
Repair using Proline 2.0 suture (Johnson & Johnson, New Brunswick, New Jersey, United States)

**Figure 3:**
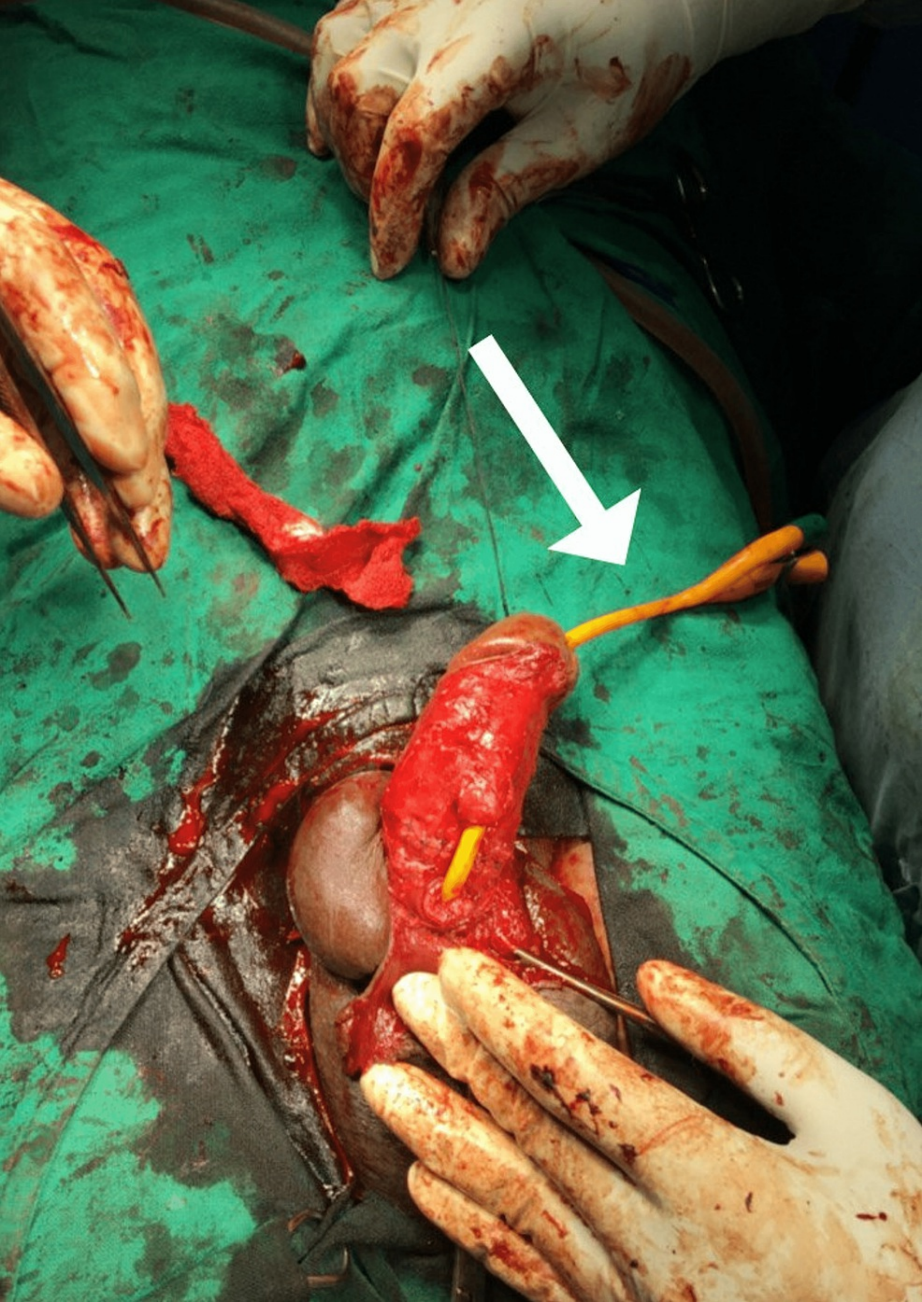
Use of Foley’s catheter that has been inserted during the surgery

The defect of the tunica albuginea of the corpora cavernosa was repaired with a Prolene 2.0 suture (Johnson & Johnson, New Brunswick, New Jersey, United States; due to its durability and to avoid any chances of reaction), and urethral reconstruction was done in two layers with a Vicryl 3.0 interrupted suture (Johnson & Johnson, New Brunswick, New Jersey, United States) and a 14-Foley catheter as shown in Figure 2 and Figure 3. 

During the hospital stay, low molecular heparin (dalteparin) and broad-spectrum antibiotics were administered. On the third postoperative day, nocturnal erections returned and were treated with diazepam. The patient improved well postoperatively without any complications. Penile oedema, a result of surgery and injury, subsided in three days, and the catheter, which was used to correct the deformity, was removed after three weeks. No recurrence has been noticed in the monthly follow-ups in the first year followed by quarterly follow-ups for the next two years.

## Discussion

Erectile tissue is organized into a columnar pattern to form the penis. One ventral corpus spongiosum and two dorsolateral corpora cavernosa, each surrounded by the tunica albuginea, form the penis. Collagen and elastin make up the bilaminar structure known as the tunica albuginea. The tunica's strength and thickness are determined by its outer layer. It varies throughout the shaft at different places and is the narrowest ventrolaterally. It has exceptional tensile strength and can withstand rupturing up to an intra-cavernous pressure of 1500 mmHg. When the penis is erect, the tunica albuginea's thickness reduces from 2 mm to 0.25 mm, and a trauma-induced spike in intracorporeal pressure during an erection might easily cause rupture [5]. The urethra runs the whole length of the corpus spongiosum. The glans penis is formed by the corpus spongiosum's distal expansion. The two corpora cavernosa are surrounded by Buck's fascia, which separates dorsally to encompass the corpus spongiosum ventrally. PF is an urgent urological ailment underreported compared to its occurrence. It is underreported due to patients' reluctance to seek medical assistance due to embarrassment, humiliation, or lack of information [6] [7].

 In 50% of instances, the "woman-on-top" position was seen, followed by "doggy style." Contrarily, Barros discovered that bilateral fractures of the corpus cavernosum and urethral lesions were more commonly related to the "doggy style" (41%), and the "man-on-top" (23%) positions in coitus, which were the most prevalent causes of PF. These investigations showed that the relative risk of PF was affected by the sexual position. Coital dysfunction, urethral fistula, penile plaque, and erectile dysfunction were side effects of the damage [8]. Prompt surgical repair is now used instead of early conservative therapy, such as cold applications, pressure dressings, catheterization, anti-inflammatory medicines, antibiotics, and erection-suppressing drugs. In 1936, Fetter and Gartman published the first surgical treatment for PF [9]. The procedure is currently considered the gold standard for treating PFs since it lowers the risk of fracture complications [9].

Up until the early 1980s, PF management had generated a great deal of controversy. Significant complications include delayed chordee, coital difficulties, arterial-venous fistulas, penile plaque, erectile dysfunction, penile curvature, nodules, and painful erections, which have been discovered to be substantially more common and can happen in up to 30-53% of patients and can be very severe. They were linked to conservative therapy. Direct incision, degloving incision, and inguinoscrotal incision are currently available surgical approach choices [10]. Excellent visibility of the three corpora is made possible by a degloving incision, although complications including an abscess, skin necrosis, or loss of feeling are possible. In cases of proximal fracture and when penile oedema is severe enough to endanger skin viability, an inguinoscrotal incision may be advised. However, wound infection runs the risk of leaving an unsightly scar as well as penile angulation. Most writers recommended degloving incision because of the high frequency of concurrent urethral damage associated with bilateral corporeal ventral PF [8]. Imaging may be necessary, especially in individuals with an unusual clinical presentation or experiencing excruciating local discomfort or swelling that makes it impossible to do a complete physical examination of the penis. The diagnosis of PF has been made using a variety of radiologic investigations [4,7].

Ultrasonography can be used to assess patients who have suffered penile injuries and determine the sort of incision necessary, saving time by preventing needless waiting. Patients who had delayed therapy had a residual fibrous area and a small curvature of the penis during erection. Regular sexual function was, nevertheless, achievable. Due to urinary extravasation and fibrous tissue, late or postponed surgery had less favorable outcomes. This fibrous tissue growth reduces the corpora's flexibility and extendibility, resulting in erectile dysfunction or penile curvature. Early diagnosis and treatment reduces the risk of postoperative erectile dysfunction. According to a study, 96% of patients who received surgical treatment and 50% of patients who received conservative treatment reported normal sexual activity with no penile curvature, the remaining 50% of the conservatively treated group experienced erectile dysfunction and penile deviation. This instance emphasizes the value of early diagnosis and surgical intervention over a conservative approach in cases of unique presentation and early surgical treatment for improved results [11].

## Conclusions

A highly uncommon occurrence called PF with urethral haemorrhage necessitates rapid surgical intervention. It most frequently happens during sexual activity. A snapping or cracking noise was the typical sign, followed by an instant detumescence and excruciating agony. An immediate surgical procedure that included penile degloving and surgical repair had a positive outcome. A Prolene 2.0 suture was used to repair the defect of the tunica albuginea of the corpora cavernosa, and urethral reconstruction was carried out with a Vicryl 3.0 suture over a 14-French Foley catheter. The patient recovered well after surgery, and no recurrence has been noticed in the monthly follow-ups in the first year followed by quarterly follow-ups for the next two years. Early surgical repair was carried out in our situation, resulting in a smooth recovery. This case report aims to further the field of medicine by offering guidance to doctors looking for experience treating this uncommon disease.
